# Representing Polar Questions

**DOI:** 10.1007/s10936-021-09814-y

**Published:** 2021-10-21

**Authors:** Ye Tian, Bob van Tiel, Élise Clin, Richard Breheny

**Affiliations:** 1 Wluper Ltd., London, UK; 2 Radboud University, Nijmegen, Netherlands; 3 Université Libre de Bruxelles, Bruxelles, Belgium; 4 University College London, London, UK

**Keywords:** Polar questions, Eye-tracking, Experimental pragmatics

## Abstract

Although the linguistic properties of polar questions have been extensively studied, comparatively little is known about how polar questions are processed in real time. In this paper, we report on three eye-tracking experiments on the processing of positive and negative polar questions in English and French. Our results show that in the early stages, participants pay attention to both positive and negative states of affairs for both positive and negative questions. In the late stages, positive and certain negative polar questions were associated with a bias for the positive state, and this bias appears to be pragmatic in nature. We suggest that different biases in mental representations reflect the hearer’s reasoning about the speaker’s purposes of enquiry.

In everyday conversation, we frequently ask and answer polar questions. Indeed, in the Switchboard Speech Act Corpus, polar questions and their answers were among the most frequent speech acts, occurring more often than all wh-questions taken together (Stolcke et al., 2000). Similarly, in a cross-linguistic corpus study, Stivers et al. (2009) found that polar questions were the most common type of questions in all of the 10 languages that they surveyed.

Polar questions can be either *positive* or *negative*, as shown for French in (1).

**Table:** 

(1)	a	Positive: Dansez-vous ?
	b	Negative: Ne dansez-vous pas ?

Certain languages make a further distinction within the class of negative polar questions depending on the surface position of the negation. For example, English distinguishes negative questions where the negation follows the auxiliary verb (*high negative*) and negative polar questions where the negation follows the subject (*low negative*) as illustrated in (2).

**Table:** 

(2)	a	High negative: Isn’t John home?
	b	Low negative: Is John not home?

Theories about the semantics of questions fall into at least two categories. The *partition view*, on the one hand, holds that questions denote sets of answers. These sets have been variously argued to consist of all possible answers (Hamblin, 1973), all true answers (Karttunen, 1977), or all exhaustive answers (Groenendijk & Stokhof, 1984). According to the partition view, there is no semantic difference between positive (?p) and negative (?not-p) polar questions. Assuming that questions denote sets of possible answers, both ?p and ?not-p thus refer to the set {p, ~p}.

The *proposition–abstraction view*, on the other hand, holds that questions denote abstractions from propositions (e.g., Ginzburg & Sag, 2000; Hausser, 1983; Krifka, 2001). Polar questions, on this view, denote nullary functions, thus being akin to assertions. Positive and negative assertions express the propositions p and ~p, respectively. Analogously, the proposition–abstraction view predicts that ?p and ?not-p have different denotations, roughly corresponding to {p} and {~p}.

Some semantic theories of polar questions also consider the dynamic potential of polar questions. Roelofsen and Farkas (2014) argue that positive and negative polar questions, while both denoting the set {p, ~p}, *highlight* different propositions. Highlighted propositions are more accessible for future anaphoric reference. According to Roelofsen and Farkas, ?p highlights the proposition p while ?not-p highlights the proposition ~p. Krifka (2013) agrees with the former claim, but argues that ?not-p introduces both p and ~p as discourse referents, with the salience of p depending on context.

Van Rooy and Šafářová (2003) argue that positive and negative polar questions are semantically identical, and that the difference between these question types lies in their pragmatic function. For positive polar questions, the utility of the positive answer is greater than the utility of the negative answer. For negative polar questions, it is the other way around. Therefore, according to Van Rooy and Šafářová, positive questions bias positive answers and negative questions bias negative answers.

There has been a fruitful debate in semantics and pragmatics aimed at describing and explaining in which respects the different forms of polar questions differ in terms of their meaning and use (e.g., Ginzburg & Sag, 2000; Krifka, 2013; Ladd, 1981; Roelofsen & Farkas, 2014; Romero & Han, 2004). However, there has been little work on discovering the online processing of different types of polar questions. This study aims to explore this topic using three eye-tracking studies on the comprehension of different types of polar questions in English and French.

## Processing Polar Questions

The goal of our eye-tracking studies was to determine how polar questions are processed and how their cognitive processing is affected by the form of the question (positive, high-neg, or low-neg in English; positive or negative in French). In particular, we were interested in seeing whether positive and negative questions make different propositions cognitively salient, as several of the aforementioned semantic theories suggest.

In order to answer this research question, we carried out a series of visual-world eye-tracking tasks. In these tasks, participants were presented with visual scenes containing relevant objects while listening to linguistic stimuli (e.g., Altmann & Kamide, 2009; Arai et al., 2007; Breheny et al., 2013; Kamide et al., 2003; Snedeker & Trueswell, 2004).

We used the same task as Tian et al. (2016), who studied the interpretation of positive and negative assertions with different prominent Questions Under Discussion (QUDs). The results of their study provide a useful baseline against which to compare the results that we will report for polar questions. Hence, we discuss the study of Tian and colleagues in some more detail.

Tian and colleagues asked participants to listen to positive and negative assertions in simple or cleft forms:

**Table:** 

(2)	a	Matt has shut his dad’s window
	b	Matt hasn’t shut his dad’s window
	c	It is Matt who has shut his dad’s window
	d	It is Matt who hasn’t shut his dad’s window

While listening to these assertions, participants were presented with four images. One of these images‒the *target*‒matched the content of the sentence, e.g., it showed a shut window in the case of (2a) and an open window in the case of (2b); another image‒the *competitor*‒mismatched with the content of the sentence, e.g., it showed an open window in the case of (2a) and a closed window in the case of (2a). The remaining two images were *distractors*, showing, e.g., a sliced pie and an unsliced pie.

Tian and colleagues found that when processing simple positive assertions, participants’ attention was shifted to the target image as soon as the verb was encountered. When processing simple negative assertions, however, participants’ attention was drawn toward both the target and the competitor image for a few hundred milliseconds before being shifted to the target. No difference was found between processing cleft positives and negatives.

Tian and colleagues argue that processing assertions out of the blue triggers the representation of different pragmatic contexts. The sentences in their experiment, when uttered out of the blue, evoke different QUDs. In particular, the sentences in (2a-b) tend to be construed as answers to a positive polar question such as ‘Has Matt shut his dad’s window?’, while the sentences in (2c-d) tend to be construed as answers to a wh-question such as ‘Who has shut his dad’s window?’ for (2c), and ‘Who hasn’t shut his dad’s window?’ for (2d).

## Our Study

In our study, participants listened to polar questions and responses, such as:

**Table:** 

(3)	a	‘Has John ironed his father’s shirt?’
		‘Yes, he has.’ / ‘No, he hasn’t.’
	b	‘Hasn’t John ironed his father’s shirt?’
		‘Yes, he has.’ / ‘No, he hasn’t.’

While listening to these question–answer pairs, participants saw four images: one image showed an ironed shirt (the p image), one image showed a crumpled shirt (the ~p image), and the other two images were distractors that were included to ensure that participants were unable to predict the upcoming content of the question.

We wanted to determine whether, upon hearing a polar question, participants fixate on both the p and ~p image, or whether they fixate primarily on only one of these images. The former is more in line with the partition view; while the latter is more consistent with the proposition–extraction view, as well as the proposals of Roelofsen and Farkas (2014), Krifka (2013), and Van Rooy and Šafářová (2003). Specifically, according to the proposition–extraction view, positive polar questions should be associated with fixations to the p image; negative polar questions with fixations to the ~p image.

In addition, we wanted to see how quickly participants would fixate on either the p or ~p image. For positive assertions, participants fixate on the correct image from the verb onwards. Hence, if polar questions are essentially assertions, as the proposition–extraction view proposes, the same should hold for polar questions. However, if the association between positive polar questions and the p image, and between negative polar questions and the ~p image is pragmatic in nature, we might expect a delay, as is generally found for pragmatic inferences (e.g., Bott & Noveck, 2004).

In Exp. 1, participants merely listened to these question–answer pairs, and answered comprehension questions to ensure that they were paying attention. In order to address concerns that this experimental setup was not sufficiently engaging, in Exps. 2 and 3, participants had to select the right display based on the answer to the question. Exps. 1 and 2 tested polar questions in English; Exp. 3 tested polar questions in French. By extending the scope to French, we are able to estimate, at least to some extent, the generalisability of our findings for English. In addition, since French only has one type of negative polar question, we were able to determine whether the results for English were influenced by the presence of two forms of negative polar questions.

## Experiment 1

The aim of Exp. 1 is to investigate the dynamic changes of visual attention while processing three types of English polar questions (positive, high-neg and low-neg) followed by answers.

### Participants

40 native English speakers were recruited from University College London via an online pool of participants. They all had uncorrected or corrected to normal vision. They were paid four pounds for their participation.

### Materials

The materials consisted of 42 question–answer pairs, such as the ones shown in (3). The questions always contained binary predicates, i.e., predicates whose negation corresponds to a well-defined situation, such as ‘ironed the shirt’, ‘opened the window’, and ‘broke the eggs’. See the “Appendix” for a list of the materials. In between the verb and the object, a genitive (e.g., ‘his father’s’) was included to allow for the time needed for anticipating referents before disambiguating information was available (Altmann & Kamide, 2007; Barr, 2008).

Each question had three forms: positive, high-neg, and low-neg. There were 8 lists containing 1 form of each of the 42 questions. Each list consisted of 14 positive questions, 14 high negative questions, and 14 low negative questions. These lists were supplemented with another 14 positive questions, thus ensuring an equal number of positive and negative questions. Since the latter 14 questions were only tested in one condition, they will not be included in the analyses below. Half of the questions in each condition were confirmed (i.e., were answered with ‘yes’); the other half rejected (i.e., were answered with ‘no’). The order of presentation was randomised across the eight lists.

**Fig. 1:**
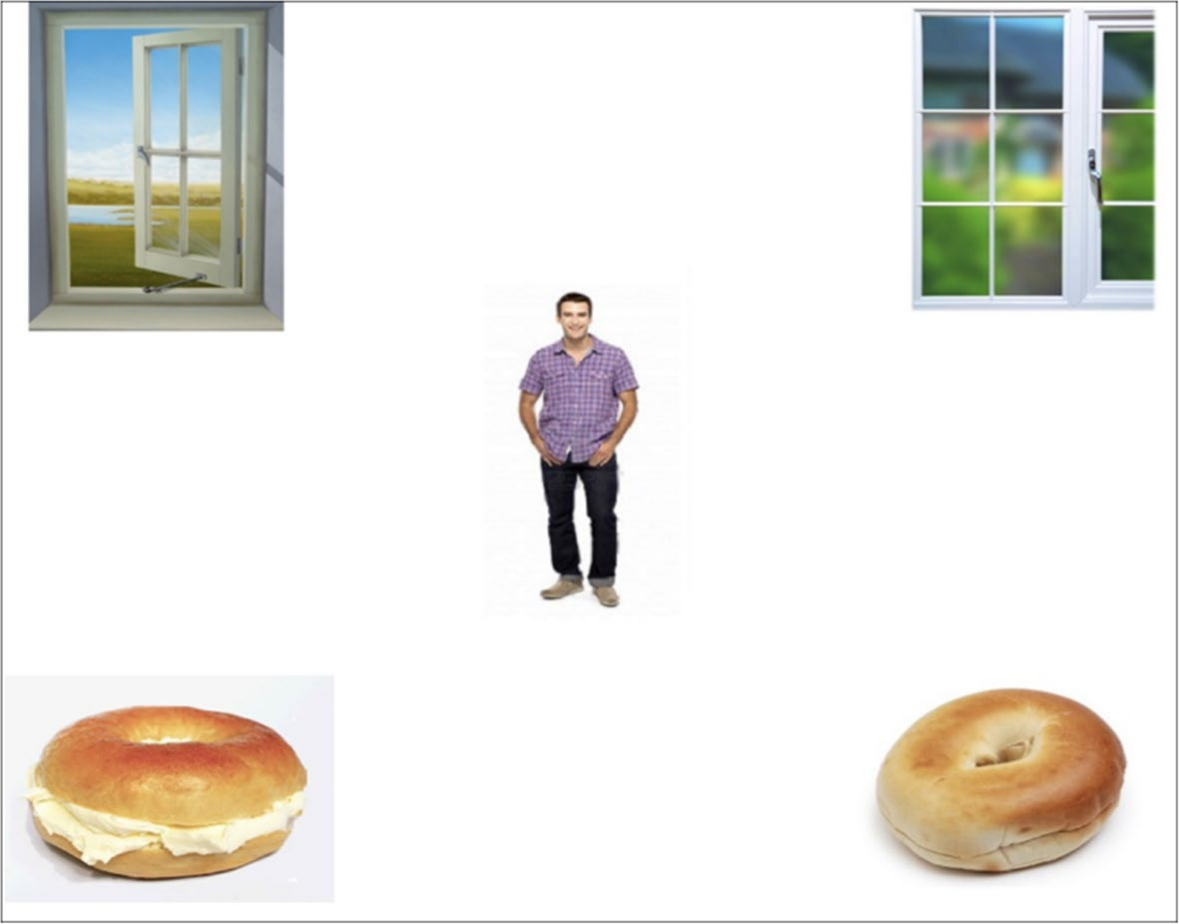
Example visual scene

Each question–answer pair was associated with a visual scene consisting of five images (see Fig. 1): in the center, an image of a person matching the gender of the person in the question, and, in the four corners, two *target* images representing p and ~p (e.g., an open window and a closed window), and two *distractors*, which were images of an item in two states (e.g., a plain bagel and a bagel with cream cheese). These distractors were included so that the participants could not predict the verb before hearing it. The critical images and the distractors all measured 250 × 250 pixels. The locations of the target and distractor images were randomised between the eight lists.

Sentences were recorded by a male speaker of South-East British English. There was a pause of 1.5 s between the question and the answer. The speaker was instructed to read all sentences with natural intonation. The average duration of the individual word regions that were identical across the three question forms (e.g., ‘ironed’, ‘his’, ‘father’s’, and ‘shirt’ in the example above) never differed by more than 20 ms. Paired *t*-tests indicated that there were no significant differences in word duration across conditions (all *p*’s > 0.47).

### Procedure

The experiment was conducted using the E-Prime software and a Tobii T300 eye-tracker. Participants were calibrated at the beginning of the experiment using a nigh-point display. Before each trial, there was a fixation cross in the centre of the screen, and participants’ eye gaze had to be fixed on this point for a continuous 1.5 s before the trial started. Then a scene of five images (as described above) appeared for one second before the audio started. Participants were instructed to listen and look at the scene. A third of the trials were followed by a comprehension question to ensure that participants paid attention to the audio.

### Data Treatment

We analysed fixations on the p and ~p images against the unfolding audio stimuli. For the analysis of eye movements, word region boundaries were offset by 200 ms to allow for the time it takes to launch eye movements (Hallett, 1986). Fixations that landed within the coordinates of the five images were analysed against key time periods (word windows and proportions of the silence window after the question). Any fixations that were deemed invalid due to blinking or head movements were removed. Any fixations shorter than 80 ms were excluded, as extremely short fixations are often due to false saccade planning (Rayner & Pollatsek, 1989). We analysed and compared fixations to the target and competitor images. Hence, we ignored fixations to the distractors, since we do not have any predictions regarding these. Data treatment and analysis methods were the same as in the study of Tian et al. (2016) which is discussed in the introduction.

### Results and Discussion

**Fig. 2:**
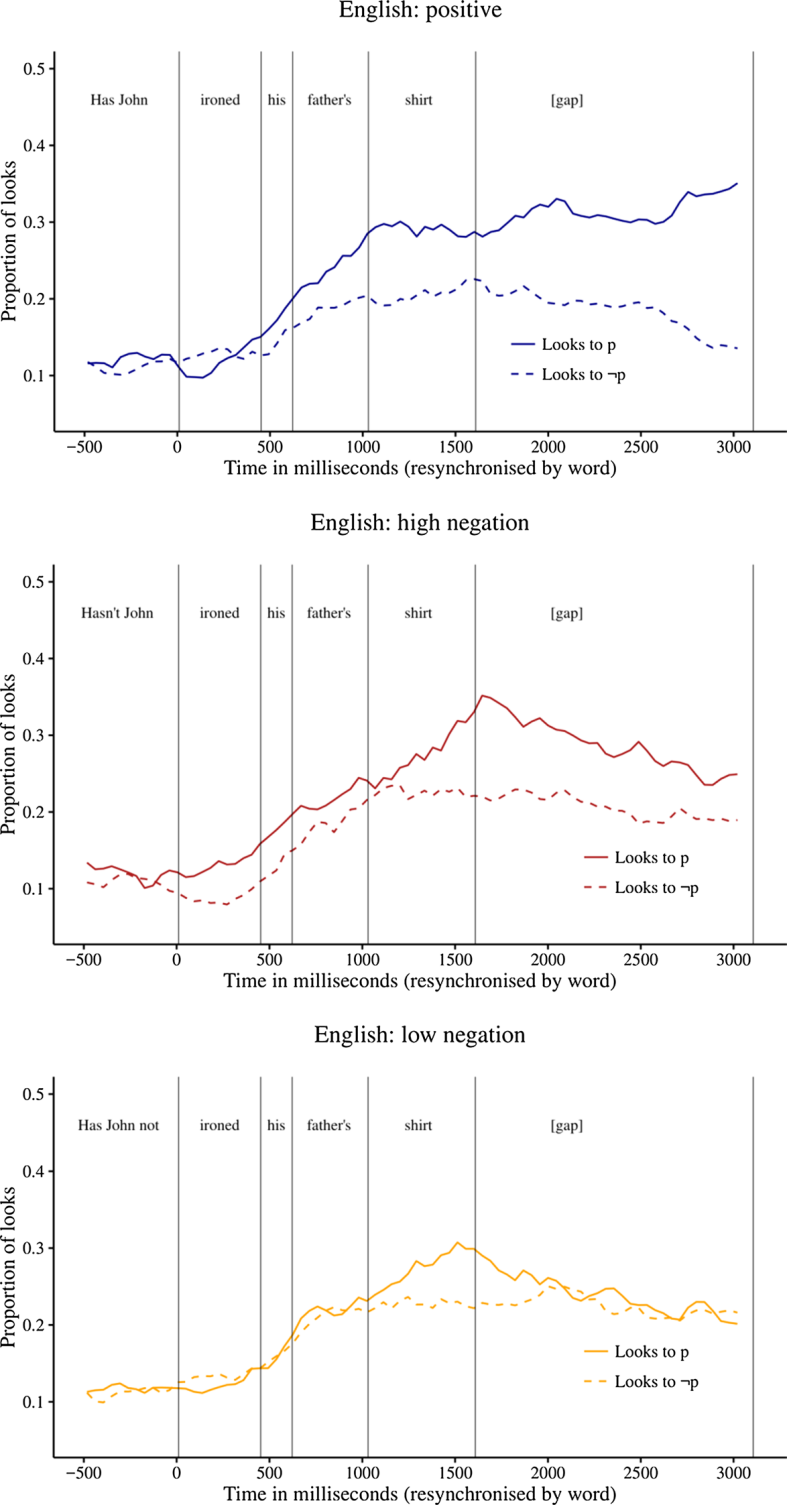
Proportions of looks to the p and ~p images in Exp. 1

Figure 2 shows the proportions of looks to the p and ~p images during the question phase and subsequent 1.5 s gap‒which is divided into two halves‒for each of the three question forms. Since each experimental item differs in the location of the onsets and offsets of each word in the audio stream, the curves in the graphs are resynchronised at the onset of each word.

The results show that participants, upon hearing a positive or negative polar question, fixate on both the p and ~p images from the verb region (e.g., ‘ironed’) onwards. In this respect, polar questions differ from positive assertions. Recall that Tian et al. (2016) observed that, upon hearing a simple assertion p, participants solely fixate on the p image from the verb onwards, largely ignoring the ~p image.

In the positive condition, a bias towards the p image emerged, starting in the genitive region (e.g., ‘father’s’) and persisting into the gap region. In the high-neg condition, the bias was similar but less prominent. In the low-neg condition, there was also a mitigated and less persistent bias towards the p image, occurring in the noun region (e.g., ‘shirt’).

In order to test whether these biases were statistically significant, we calculated the natural log ratio of the probability of looks to the p image over looks to the ~p image. This measure is symmetrical around 0, meaning that a positive log ratio corresponds to a bias towards the p image and a negative log ratio to a bias towards the ~p image. We conducted mixed linear regression analyses to determine if the log ratios differed reliably from 0. The results of these analyses are shown in Table 1.

**Table 1:** Natural log ratio of the probability of looks to the p over ~p image

		ironed	his	father’s	shirt?	[gap-1]	[gap-2]
	ln	− .09	.10	.22	.36	.40	.63
Pos	(df) *t*	(41) 0.1	(41) 0.5	(26) 1.8	(41) 3.6	(38) 2.6	(41) 4.3
	*p*	.90	.60	.08	.00	.00	.00
	ln	.37	.09	.16	.14	.36	.36
H-Neg	(df) *t*	(31) 1.8	(24) 0.6	(38) 1.0	(40) 1.9	(29) 3.4	(25) 3.2
	*p*	.08	.55	.33	.07	.00	.00
	ln	− .11	− .01	.13	.15	.15	− .01
L-Neg	(df) *t*	(30) 0.4	(517) 0.3	(39) 0.7	(39) 2.4	(31) 0.1	(40) 0.9
	*p*	.67	.77	.47	.02	.14	.95

Positive values correspond to bias to the p image; negative values to bias to the ~p image. The corresponding *t* and *p* values indicate whether the value is significantly different from 0, based on a mixed linear regression analysis

In the positive condition, there was a significant bias towards the p image from the genitive onwards. In the high-neg condition, the bias emerged in the noun region. In the low-neg condition, the bias also emerged in the noun region, but disappeared immediately afterwards.

In order to determine if the log ratio differed across conditions, we conducted, for each pair of conditions, mixed linear regression analyses predicting log ratio based on condition. In these analyses, we included random intercepts and slopes for participants and items (Barr et al., 2013). If this analysis did not converge, we stepwise dropped the random slopes for items and participants. The alpha value was corrected for multiple comparisons and set at 0.016. The analysis thus indicated that log ratio differed significantly during the second half of the gap phase between positive and low-neg, and between high-neg and low-neg (positive vs. high-neg: *t*(34) = 2.1, *p* = 0.04, model = intercepts only; positive vs. low-neg: *t*(36) = 3.2, *p* = 0.00, model = intercepts for subjects and items; slopes for items; high-neg vs. low-neg: *t*(36) = 2.8, *p* = 0.01, model = intercepts for subjects and items; slopes for items). The positive bias was thus more persistent in the positive condition and in the high-neg condition compared to the low-neg condition; there was no significant difference between the positive and high-neg condition.

## Experiment 2

Exp. 1 showed that participants developed a bias towards the p image in all three conditions, although the amplitude and duration of this bias differed across conditions. At the same time, it is unclear how robust these findings are. In natural conversations, when we hear a polar question we must process it and then form an answer. In Exp. 1, participants did not need to answer these questions, and therefore did not need to pay close attention to the images. Indeed, the comprehension questions that followed a third of the trials targeted the content of the question–answer pair that participants heard rather than the images that they saw. For that reason, Exp. 2 uses a slightly different experimental task that is closer to the natural purpose of polar questions. Participants in this task were instructed to indicate which of the four images best represented the situation described by the question–answer pair. For example, participants who heard (4) had to select the image showing an ironed shirt.

**Table:** 

(4)	‘Has John ironed his father’s shirt?’
	‘Yes, he has.’

### Participants

40 native English speakers were recruited from University College London via an online pool of participants. All participants had normal or corrected to normal vision and were paid four pounds for their participation.

### Materials

The materials were the same as in Exp. 1.

### Procedure

Participants were instructed to identify the image that corresponded to the answer in the question–answer pair. To this end, the four images were geographically mapped to the keys ‘e’, ‘c’, ‘i’, and ‘m’ on the keyboard. These buttons were marked with coloured stickers. Participants were asked to place four fingers on these keys and not look down during the experiment. This did not pose any difficulties to the participants. Participants were familiarised with the procedure by means of four practice trials that included feedback on their performance. Other than this, the procedure was the same as for Exp. 1.

### Results and Discussion

All participants achieved an accuracy of over 85% and data from all participants were included. Filtering of eye-tracking data followed the same procedure as Exp. 1.

First, we briefly consider the behavioural data to validate the experimental task. Overall, accuracy was close to the ceiling at 94.4% correct responses. Participants were most accurate for positive questions (96.9% correct for both ‘yes’ and ‘no’ responses) and least accurate for low-neg questions (90.3% correct for ‘yes’ responses and 93.4% for ‘no’ responses). Accuracy for high-neg questions fell in between (95.8% correct for ‘yes’ responses and 93.4% correct for ‘no’ responses).

Response times, measured from onset of the answer particle, were fastest for positive questions (1282 ms for ‘yes’ answers and 1230 ms for ‘no’ answers). Response times for high-neg questions were substantially slower (1412 ms for ‘yes’ answers and 1514 ms for ‘no’ answers). Response times for low-neg questions were the slowest (1632 ms for ‘yes’ answers and 1627 ms for ‘no’ answers. All of these results accord with our prior expectations. Hence, we now consider participants’ eye movements.

**Fig. 3:**
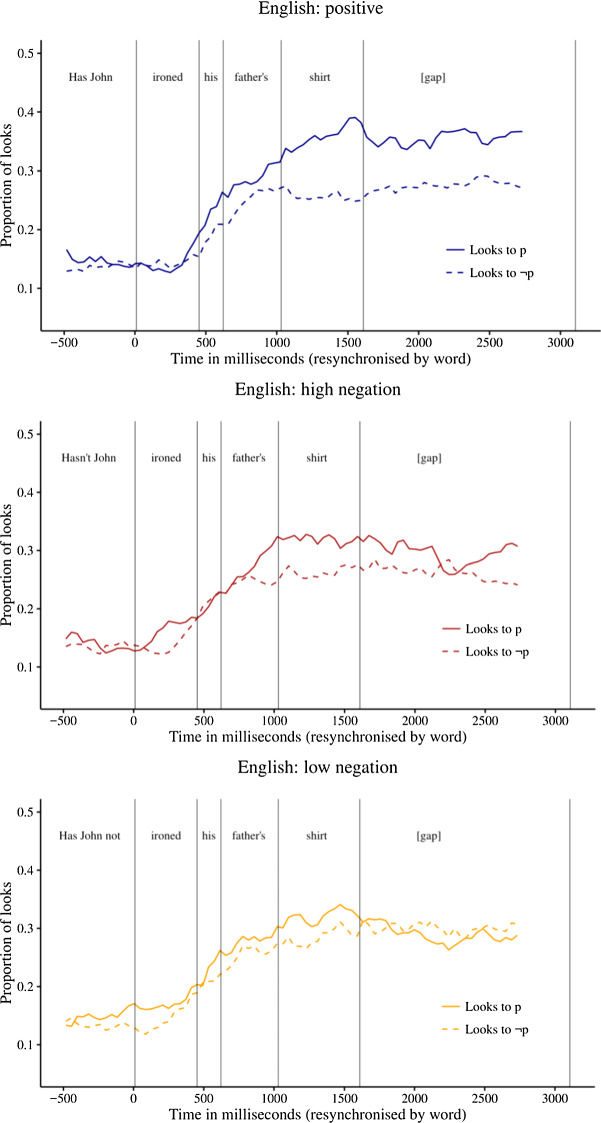
Proportions of looks to the p and ~p images in Exp. 2

Figure 3 shows the proportions of looks to the p and ~p images during the question phase and subsequent 1.5 s gap for each of the three question forms.

In the positive condition, there was a stable bias towards the p image from the noun region onwards. In the high-neg condition, there was a small bias to the p image in the noun region. Finally, in the low-neg condition, there was no clear bias to either the p or ~p image.

In order to test whether the biases were statistically significant, we calculated the natural log ratio of the probability of looks to the p image over looks to the ~p image. A positive log ratio corresponds to a bias towards the p image and a negative log ratio to a bias towards the ~p image. We conducted mixed linear regression analyses to determine if the log ratios differed reliably from 0. In these analyses, we included random intercepts for participants and items. The degrees of freedom were estimated using the “pbkrtest” package. The results of these analyses are shown in Table 2.

**Table 2:** Natural log ratio of the probability of looks to the p over ~p image

		ironed	his	father’s	shirt?	[gap-1]	[gap-2]
	ln	.03	.18	.28	.53	.49	.28
Pos	(df) *t*	(32) 0.2	(32) 1.1	(34) 1.4	(25) 3.6	(29) 2.7	(29) 1.7
	*p*	.81	.30	.16	.00	.01	.00
	ln	.10	− .03	.21	.34	.27	.22
H-Neg	(df) *t*	(27) 0.8	(26) 0.2	(32) 1.2	(32) 1.9	(32) 1.4	(25) 1.4
	*p*	.44	.84	.25	.06	.16	.18
	ln	.14	.07	.13	.14	.14	.06
L-Neg	(df) *t*	(33) 0.9	(27) 0.4	(28) 0.8	(33) 0.7	(33) 0.7	(26) 0.4
	*p*	.40	.68	.43	.47	.48	.70

Positive values correspond to bias to the p image; negative values to bias to the ~p image. The corresponding *t* and *p* values indicate whether the value is significantly different from 0, based on a mixed linear regression analysis

Like in Exp. 1, participants in Exp. 2 paid attention to both p and ~p images when hearing all types of polar questions. In this respect, polar questions differ from positive assertions, for which Tian and colleagues observed immediate fixation on the p image.

In the positive condition, there was a significant bias towards the p image in the noun region and during the first half of the gap between question and answer. In the high-neg condition, there was a marginally significant positive bias in the noun region. In the low-neg condition, there was no significant bias in either direction. In no condition was there a bias towards ~p.

In order to determine if the log ratio differed across conditions, we conducted, for each pair of conditions, mixed linear regression analyses predicting log ratio based on condition, with random intercepts and slopes for participants and items. Again, we stepwise dropped the random slopes if the models failed to converge. The alpha value was corrected for multiple comparisons and set at 0.016. This analysis indicated that the log ratio in the positive condition is numerically but not significantly higher than the low-neg condition during the noun region and during the first half of the gap (*p* = 0.03 and *p* < 0.05, model = intercepts only). All other comparisons were not significant.

The positive bias for positive and high negative questions that we found in Exp. 1 was thus confirmed in Exp. 2, although the bias was less prominent and less persistent, in particular in the high-neg condition. The bias towards the p image for low negative questions that we found in Exp. 1 was not replicated in Exp. 2.

Presumably, the reason the positive bias was less prominent in Exp. 2 is that the task forced participants to consider both the p and ~p images. Recall that, in Exp. 1, participants merely listened to the question–answer pair, whereas participants in Exp. 2 were instructed to indicate which image corresponded to the situation described in the question–answer pair. In all three conditions, this was equally likely to be the p or ~p image. It is striking, then, that even in this situation, participants had a (mitigated) bias for the p image in the positive and high-neg conditions.

## Experiment 3

The first two experiments tested the interpretation of polar questions in British-English, even though the conclusions that we would like to draw are much wider in scope. To address this, we replicated Exp. 2 in a different language: Belgian-French. Of course, even if we confirm the findings of Exps. 1 and 2 in one different language, this would not guarantee that the pattern of results is universal. It would, however, increase the experimental base on which to build more wide-ranging conclusions.

There are a couple of differences between polar questions in English and French that warrant discussion up front. First, French has three constructions that can be used to ask questions. To illustrate, the question ‘Do you dance? can be translated into French as:

**Table:** 

(5)	a	Declarative: Vous dansez?
	b	Inversion: Dansez-vous?
	c	Est-ce que: Est-ce que vous dansez?

In order to facilitate the comparison with English, and because it is the most natural form with which to ask negative questions, we tested questions that are marked by means of subject-verb inversion, as in (5b).

Second, French has no distinction between high-neg and low-neg questions (e.g., ‘Didn’t he call?’ and ‘Did he not call?’). Hence, we only tested two conditions: positive polar questions (‘Did he call?’) and negative polar questions (‘Didn’t he call?’).

Third, the response particle system in French is less ambiguous than in English. In particular, when responding to a negative question, ‘Yes’ and ‘No’ in English are variously used to confirm or reject the corresponding question. In our experiment, however, we always used ‘Yes’ to confirm and ‘No’ to reject questions, and the intended meaning was disambiguated in the continuation (e.g., ‘Yes, he has’ vs. ‘No, he hasn’t’). In French, by contrast, ‘Si’ unambiguously confirms and ‘Non’ unambiguously rejects negative questions (Noveck et al., 2021). Since we are only interested in the question phase, however, this difference is not particularly relevant to our purposes.

In the next section, we describe the experiment in more detail.

### Participants

42 native French speakers were recruited from Université Libre de Bruxelles via an online pool of participants. They all had uncorrected or corrected to normal vision. They were paid four euros for their participation.

### Materials

The 42 question–answer pairs that were tested in Exps. 1 and 2 were translated into French and recorded by a native speaker of Belgian-French. Since French does not have the genitive before its argument (e.g., ‘his father’s shirt’ vs.‘la chemise de son père’), we instead placed a temporal adverb between the verb and the object. Participants thus listened to sequences such as:

**Table:** 

(6)	‘Jean a-t-il repassé cet après-midi sa chemise?’
	‘Oui, il l’a repassé.’ / ‘Non, il ne l’a pas repassé.’

The average duration of the individual word regions that were identical across the three question forms (e.g., ‘repassé’, ‘cet’, ‘après-midi’, ‘sa’, ‘chemise’) never differed by more than 30 ms. Paired *t*-tests indicated that there were no significant differences in word duration across conditions (all *p*’s > 0.35).

### Procedure

The procedure was the same as in Exp. 2.

### Results and Discussion

First, we briefly consider the behavioural data to validate the experimental task. Accuracy was close to ceiling at 96.3% correct responses. Participants were more accurate for negative questions (98.4% correct for ‘yes’ responses and 95.0% for ‘no’ responses) than for positive questions (98.2% correct for ‘yes’ responses and 93.5% for ‘no’ responses). Recall that, for English, we observed the lowest accuracy for ‘yes’ responses to negative questions; for French, accuracy was the highest in this condition. This difference is likely caused by the presence of French ‘si’ as an unambiguous way of referring to a positive state of affairs, whereas in English ‘yes’ is ambiguous between referring to a positive or negative state of affairs.

Response times were also numerically faster for negative questions (1425 ms for ‘yes’ answers and 1582 ms for ‘no’ answers) compared to positive ones (1418 ms for ‘yes’ answers and 1603 ms for ‘no’ answers). However, the difference between positive and negative questions was extremely small. Again, these observations accord with our prior expectations. Hence, we now turn to the eye-tracking data.

**Fig. 4:**
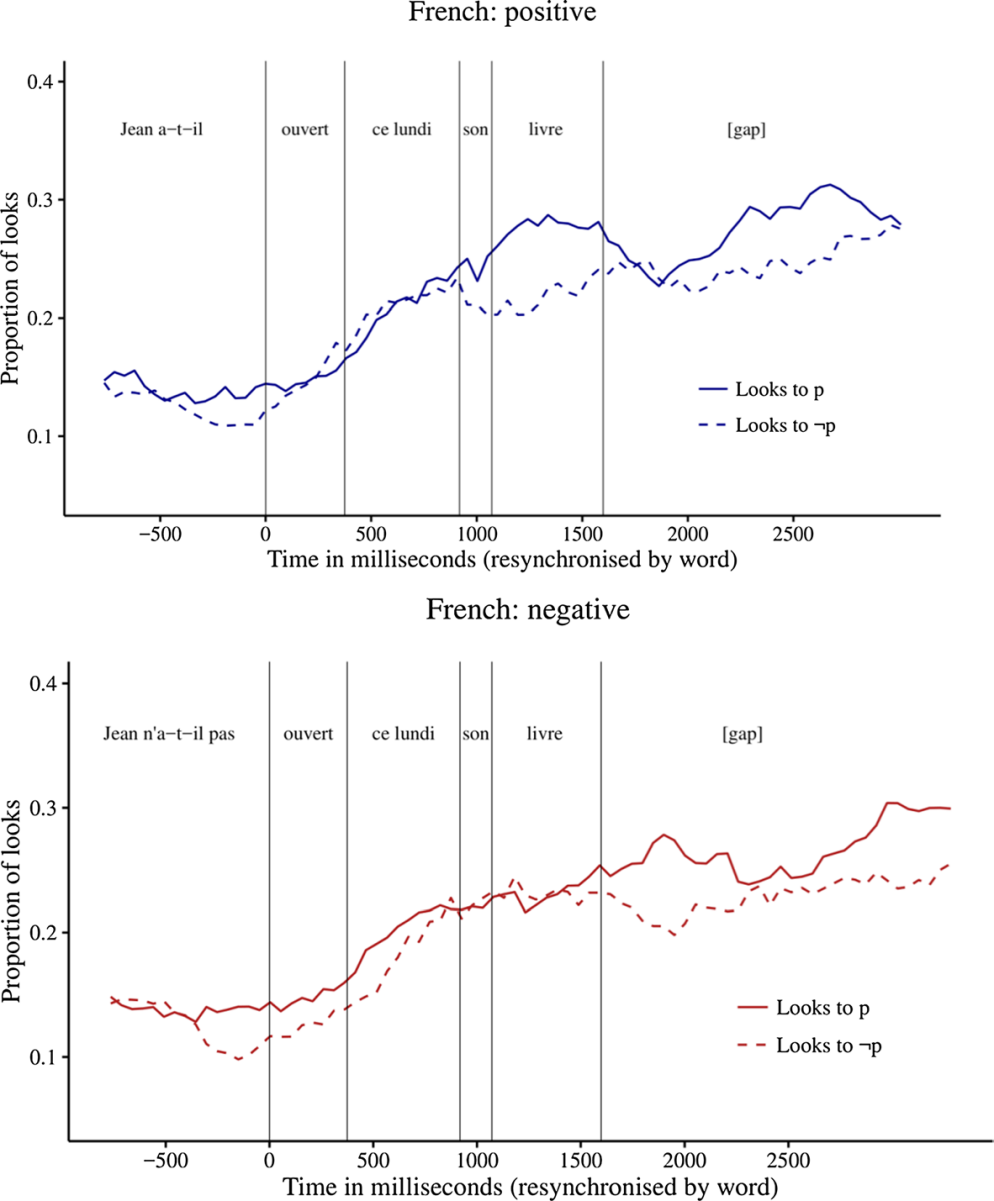
Proportions of looks to the p and ~p images in Exp. 3

Figure 4 shows the proportions of looks to the p and ~p images during the question phase and subsequent 1.5 s gap for each of the three question forms. In the positive condition, there was a stable bias towards the p image in the noun region and in the second half of the gap between question and answer. In the negative condition, there was a small bias to the p image in the gap region.

To test whether the biases were statistically significant, we calculated the natural log ratio of the probability of looks to the p image over looks to the ~p image. We conducted mixed linear regression analyses to determine if the log ratios differed reliably from 0. In these analyses, we included random intercepts and slopes for participants and items. If this analysis did not converge, we stepwise dropped random slopes for items and participants. The results of these analyses are shown in Table 3.

**Table 3:** Natural log ratio of the probability of looks to the p over ~p image

		repassé	cet après-midi	sa chemise?	[gap-1]	[gap-2]
		ironed	this afternoon	his shirt?	[gap-1]	[gap-2]
	ln	.14	− .01	.19	.05	.21
Pos	(df) *t*	(27) 1.0	(35) 0.1	(21) 3.1	(31) 0.7	(32) 2.3
	*p*	.34	.92	.00	.49	.03
	ln	.18	.06	− .00	.16	.11
Neg	(df) *t*	(22) 2.1	(33) 0.3	(27) 0.1	(24) 1.9	(32) 0.9
	*p*	.05	.80	.95	.06	.36

Positive values correspond to bias to the p image; negative values to bias to the ~p image. The corresponding *t* and *p* values indicate whether the value is significantly different from 0, based on a mixed linear regression analysis

Again, participants initially paid attention to both p and ~p images in both positive and negative conditions. In the positive condition, there was a significant bias towards the p image in the noun region and during the second half of the gap between question and answer. In the negative condition, there was a marginally significant positive bias in the verb region and during the first half of the gap between question and answer.

In order to determine if the log ratio differed across conditions, we conducted mixed linear regression analyses predicting log ratio based on condition (positive or negative), with random intercepts and slopes for participants and items. Again, we stepwise dropped the random slopes if the models failed to converge. The alpha value was corrected for multiple comparisons and set at 0.016. This analysis indicated that, in the noun region, the log ratio in the positive condition was numerically but not significantly higher than in the negative condition (*t*(79) = 2.3, *p* = 0.03, model = intercepts only).

The results for positive questions are similar to the results for positive questions in Exps. 1 and 2, showing a mitigated bias for the p image from the noun region onward. The results for negative questions are more difficult to interpret. In particular, there was a marginally significant positive bias already in the verb region. Given that we did not find such an early bias in either of the previous experiments, and given that the effect was only marginally significant, we will not consider this effect in more detail. In addition, there was a marginally significant positive bias in the first half of the gap between question and answer, which is in line with the results of Exps. 1 and 2.

In the next section, we discuss the consequences of these findings for semantic theories of polar questions.

## General Discussion

We set out to determine how positive and negative polar questions differ from a cognitive viewpoint by observing visual attention in their online processing. To that end, we conducted three eye-tracking studies in English and French. In each of these studies, participants listened to question–answer pairs such as the following:

**Table:** 

(8)	a	‘Has John ironed his father’s shirt?’
		‘Yes, he has.’ / ‘No, he hasn’t.’
	b	‘Hasn’t John ironed his father’s shirt?’
		‘Yes, he has.’ / ‘No, he hasn’t.’
	c	‘Has John not ironed his father’s shirt?’
		‘Yes, he has.’ / ‘No, he hasn’t.’

While listening to these dialogues, participants saw visual scenes containing, inter alia, an image of an ironed shirt (the p image) and an image of a crumpled shirt (the ~p image).

In Exp. 1, participants merely listened to these dialogues while watching visual scenes, occasionally answering comprehension questions to determine whether they were paying attention. In Exps. 2 and 3, participants were instructed to indicate which image corresponded to the answer in the question–answer pair. Exps. 1 and 2 tested polar questions in English (positive, high-neg, and low-neg); Exp. 3 tested polar questions in French (positive and negative).

The results of the three experiments were largely concurrent. In all conditions, participants paid increased attention to both p and ~p starting soon after hearing the verb, suggesting that participants consider both alternative states of affairs when processing a polar question. In this respect, polar questions differ from positive assertions, as Tian et al. (2016) found that when hearing positive assertions, participants paid no extra attention to the ~p image in any region. The results for polar questions also differ from what Tian and colleagues observed for negative assertions, namely initial fixation on the p image followed by fixation on the (correct) ~p image. Table 4 conveniently compares the results that Tian and colleagues found for simple assertions to the results that we observed for polar questions.

**Table 4:** Summary of the results of this study in comparison with the results of Tian et al. (2016) for positive and negative assertions

	Initial fixation	Later fixation
Positive assertion (Tian et al., 2016)	p	p
Simple negative assertion (Tian et al., 2016)	p	~p
Positive polar question (this study)	{p, ~p}	p
High-negative polar question (this study)	{p, ~p}	p
Low-negative polar question (this study)	{p, ~p}	{p, ~p}

In later regions, positive questions were associated with a bias towards the p image from the noun region (e.g., ‘shirt’ in the example above) onwards. A smaller and less persistent bias towards the p image was observed for high-neg questions in English and for negative questions in French. Low-neg questions did not have a clear bias pattern. Although Exp. 1 observed a marginally significant bias towards the p image during the noun region, this effect was not replicated in Exp. 2. A negative bias (i.e., a bias towards ~p) was never observed.

Assuming that semantic representations straightforwardly map onto cognitive representations (e.g., Lidz et al., 2011), the results are particularly problematic for all approaches that assume that negative polar questions either denote or make salient a negative proposition. Indeed, in this respect, the processing of negative polar questions differs from the processing of negative assertions, for which Tian and colleagues found a delayed but robust bias for the ~p image.

By contrast, the results for the earlier region are in accordance with the partition view, according to which positive and negative polar questions make both the positive and negative states of affairs salient. At the same time, the results for the later regions are more difficult to reconcile with any of the theoretical approaches outlined in the introduction. The finding that positive polar questions were associated with fixations to the p image are at least in line with the idea that these questions make the positive proposition salient. But the observation that negative polar questions were also associated with a positive bias is not predicted by any of the theoretical approaches. The only theory that potentially can be reconciled with this observation is Krifka’s (2013).

Krifka’s approach is couched within the partition view. However, he argues that positive polar questions make the positive proposition salient, whereas negative polar questions can make both the positive and negative proposition salient, and that the salience of the positive proposition varies with the context. One possibility is that the positive proposition was generally highly salient in our experiment, perhaps because half of the trials involved a reference to the positive proposition.

Here, we also offer a more speculative explanation. We propose that during the online processing of polar questions, participants represent the questioner’s ‘state of mind’ or her purpose of enquiry. A questioner asks a positive question when she already has some positive evidence, or when she has no evidence one way or the other. In the first case, it is relevant for the addressee to evaluate the available positive evidence; in the second case, confirmation bias drives us to search for confirming evidence first. Thus, cognitive attention to the state p is relevant when someone asks a positive question.

We argue that representing the ‘state of enquiry’ also accounts for the results of high-neg and low-neg conditions in English, and the negative condition in French. A questioner asks a high-neg question when she has an epistemic bias towards p (Romero & Han, 2004). There may or may not be any evidence present, but if there is any it is for ~p (clashing with the epistemic bias). We cannot speculate on how the positive epistemic bias and the optional negative evidential bias interact with each other, but our result (a weak positive bias) is not incompatible with representing such a mental state.

The case of the low-neg questions in English and negative questions in French is slightly more mysterious. A speaker asks a low-neg question when there is some evidence for ~p. She may or may not have an epistemic bias, but if there is one it is for p (clashing with the evidence). A negative question in French can have the function of a high-neg or a low-neg English question, depending on the context and the question prosody. Given such states of enquiry, we might expect to observe a negative bias, but that was never observed. Why? One possibility is that some low-neg questions, especially in British English, can carry an epistemic bias for p. For example, ‘Is there not some call on judges to be just a little more respectful perhaps when they’re dealing with cases like this?’ (British National Corpus HUV S broadcast discussion). This question implies that the speaker expects p. The other possibility is that the optional positive epistemic bias counter-balanced the negative evidential bias (Sudo, 2013); just like for high-neg questions, the optional negative evidential bias reduced the degree of positive epistemic bias.

Overall, we argue that gaze biases in the late regions represent pragmatic processing: we represent the questioner’s ‘state of enquiry’. This conclusion rests on the assumption that semantic processing occurs relatively early, whereas the preferences that we observed often emerged more than a second after the question type was disambiguated. We should acknowledge, however, that, while there is evidence in favour of mapping early and late preferences to semantic and pragmatic processing (e.g., Bott & Noveck, 2004; Huang & Snedeker, 2009, 2011), there is also some evidence against this view (e.g., Degen & Tanenhaus, 2015; Grodner et al., 2010; Sun & Breheny, 2020). We should therefore emphasise that our conclusion is contingent on the assumption that semantic processing occurs early.

## Conclusion

This paper investigated the online processing of polar questions, and the properties that may influence the mental representation of polar questions. Through three visual world eye-tracking experiments, we found that in both English and French, the mental representations of polar questions involve representing both p and ~p, which is different from what Tian et al. (2016) observed for assertions. Different forms of polar questions are represented differently, and the difference is not symmetrical by polarity. We observed positive biases in positive polar questions and to a lesser extent in high-neg questions in English and negative questions in French. English low-neg questions showed no clear bias. We concluded that the different biases in mental representations reflect the hearer’s reasoning about the speaker’s purposes of enquiry.

## Appendix

### Materials Used in Exps. 1 and 2

All experimental sentences tested in Exps. 1 and 2 appear in three conditions: positive, high-neg and low-neg. For example:

**Table:**

(9)	a	*Positive*: Has Anne closed her mom’s umbrella?
	b	*High-neg*: Hasn’t Anne closed her mom’s umbrella?
	c	*Low-neg*: Has Anne not closed her mom’s umbrella?

The following list of experimental sentences only shows the positive forms.

**Table:**

(10)	Has Amy finished her cousin’s jigsaw puzzle?
	Has Andrew lit his auntie’s candle?
	Has Daniel emptied his mum’s saucepan?
	Has Dave cleaned his wife’s wellies?
	Has Edward turned on his friend’s tap?
	Has Emma rolled up her son’s sleeves?
	Has Grant sliced his chef’s cucumber?
	Has Ian cooked his sister’s spaghetti?
	Has Lilly cracked her sister’s egg?
	Has Lucy framed her sister’s photo?
	Has Mary folded her friend’s deck chair?
	Has Nathan emptied his colleague’s truck?
	Has Rita screwed up her sister’s wrapping paper?
	Has Tracy sliced her mom’s bread?
	Has Ben broken his friend’s pencil?
	Has Bill tied his son’s shoe laces?
	Has Dave peeled his sister’s banana?
	Has James blown up his cousin’s balloon?
	Has Jessica picked up her husband’s phone?
	Has John ironed his father’s shirt?
	Has Justin erased his teacher’s blackboard?
	Has Lee sealed his boss’s envelope?
	Has Mike folded his wife’s scarf?
	Has Nick decorated his friend’s Christmas tree?
	Has Paul dried his auntie’s tomatoes?
	Has Sophie closed her sister’s drawer?
	Has Susan mended her son’s jeans?
	Has Susan rolled up her friend’s yoga mat?
	Has Aiden washed his dad’s car?
	Has Anna closed her mom’s umbrella?
	Has Chris fastened his son’s zip?
	Has Eva scratched her brother’s CD?
	Has Gavin opened his son’s can?
	Has James inflated his brother’s tyre?
	Has Jim opened his friend’s padlock?
	Has John opened his friend’s book?
	Has John turned off his uncle’s TV?
	Has Linda iced her auntie’s cupcake?
	Has Lucas turned off his wife’s light bulb?
	Has Matt shut his dad’s window?
	Has Tina emptied her mom’s jug?
	Has Zoey cut her sister’s cake?

### Materials Used in Exp. 3

All experimental sentences tested in Exp. 3 appear in two conditions: positive and negative. For example:

**Table:**

(11)	a	*Positive*: Jean a-t-il repassé cet après-midi sa chemise?
	b	*Negative*: Jean n’a-t-il pas repassé cet après-midi sa chemise?

The following list is shown in positive format:

**Table:**

(12)	Gérard a-t-il coupé cette matinée le concombre ?
	Lucas a-t-il éteint cette nuit l’ampoule ?
	Rita a-t-elle déchiré ce weekend le papier cadeau ?
	Gaston a-t-il ouvert ce soir une cannette ?
	David a-t-il pelé ce matin sabanane ?
	Louis a-t-il fermé ce midi l’enveloppe ?
	Philippe a-t-il gonflé ce midi son pneu ?
	Jean a-t-il ouvert ce lundi son livre ?
	Bill a-t-il lacé ce matin ses chaussures ?
	Grégoire a-t-il ouvert ce jeudi le cadenas ?
	Philippe a-t-il gonflé cet aprés-midi le ballon ?
	André a-t-il allumé ce soir la bougie ?
	Matthieu a-t-il fermé ce matin la fenêtre ?
	Marine a-t-elle plié ce matin son écharpe ?
	Sophie a-t-elle fermé cette fois le tiroir ?
	Daniel a-t-il vidé ce mercredi la casserole ?
	Emma a-t-elle remonté cet aprés-midi ses manches ?
	Théo a-t-il lavé ce weekend la voiture ?
	Gaétane a-t-elle vidé ce midi la carafe ?
	Nicolas a-t-il décoré cette année le sapin de Noël ?
	Christophe a-t-il fermé ce matin sa braguette ?
	Marie a-t-elle replié ce midi le transat ?
	Jean a-t-il éteint cette nuit la télévision ?
	Nathan a-t-il vidé cet automne le camion ?
	Jessica a-t-elle répondu ce midi au téléphone ?
	Eva a-t-elle rayé cet aprés-midi son CD ?
	Amélie a-t-elle fini cet hiver le puzzle ?
	Ivan a-t-il cuisiné ce midi des spaghettis ?
	Ben a-t-il cassé cet aprés-midi son crayon ?
	Lucie a-t-elle encadré cet été la photo ?
	Justin a-t-il effacé ce matin le tableau ?
	Zoé a-t-elle coupé cette aprés-midi le cake ?
	Élise a-t-elle cassé ce matin l'œuf ?
	Justine a-t-elle coupé ce midi le pain ?
	Edouard a-t-il ouvert ce printemps le robinet ?
	Anna a-t-elle fermé cette fois le parapluie ?
	Paul a-t-il séché cet hiver les tomates ?
	Suzanne a-t-elle réparé ce matin son pantalon ?
	Lisa a-t-elle glacé cet aprés-midi le cupcake ?
	David a-t-il nettoyé ce weekend ses bottes ?
	Suzanne a-t-elle roulé ce matin son tapis de yoga ?
	Jean a-t-il repassé cet aprés-midi sa chemise ?
